# Astrocyte senescence: Evidence and significance

**DOI:** 10.1111/acel.12937

**Published:** 2019-02-27

**Authors:** Justin Cohen, Claudio Torres

**Affiliations:** ^1^ Department of Pathology and Laboratory Medicine Drexel University College of Medicine Philadelphia Pennsylvania

**Keywords:** aging, astrocyte senescence, astrogliosis, neurodegeneration, neuroinflammation

## Abstract

Astrocytes participate in numerous aspects of central nervous system (CNS) physiology ranging from ion balance to metabolism, and disruption of their physiological roles can therefore be a contributor to CNS dysfunction and pathology. Cellular senescence, one of the mechanisms of aging, has been proposed as a central component of the age dependency of neurodegenerative disorders. Cumulative evidence supports an integral role of astrocytes in the initiation and progression of neurodegenerative disease and cognitive decline with aging. The loss of astrocyte function or the gain of neuroinflammatory function as a result of cellular senescence could have profound implications for the aging brain and neurodegenerative disorders, and we propose the term “astrosenescence” to describe this phenotype. This review summarizes the current evidence pertaining to astrocyte senescence from early evidence, in vitro characterization and relationship to age‐related neurodegenerative disease. We discuss the significance of targeting senescent astrocytes as a novel approach toward therapies for age‐associated neurodegenerative disease.

## 1 INTRODUCTION

Aging is characterized as a time‐dependent deterioration in the physiological integrity of living organisms. This functional decline has become incredibly relevant in the modern era, where advances in medicine have allowed humans to live longer than ever before. In light of the economic and social impact of aging and age‐associated diseases, there has been extensive research into the underlying cellular mechanisms of aging. In fact, substandard results from clinical trials aimed at ameliorating age‐associated neurodegenerative diseases (Athauda & Foltynie, 2016; Cummings, Morstorf, & Zhong, 2014) suggest that aging is not only a risk factor for disease, but may rather be an underlying cause. In fact, the central nervous system (CNS) undergoes numerous detrimental changes as one ages including mitochondrial dysfunction, oxidative stress, and chronic inflammation (Chakrabarti et al., 2011; Gemma, Vila, Bachstetter, & Bickford, 2007; Kiecolt‐Glaser et al., 2003). Therefore, targeting the mechanisms of CNS aging may be therapeutically prudent.

**Figure 1:**
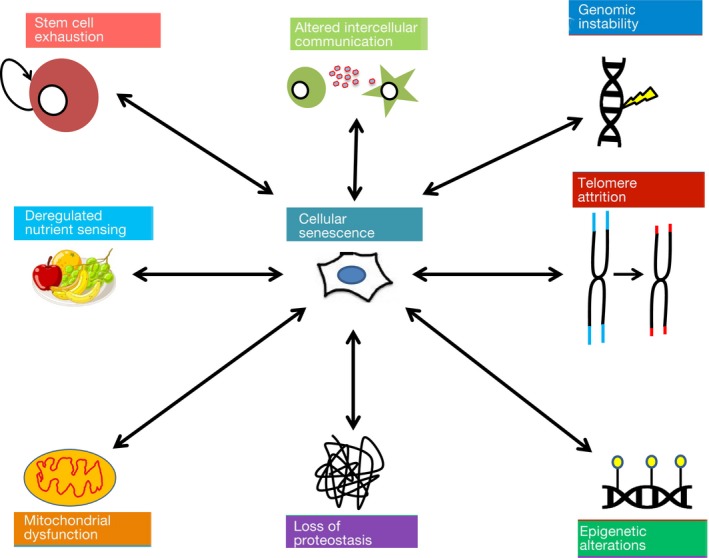
Relationship between cellular senescence and the other hallmarks of aging. Each hallmark of aging shares a tight relationship with cellular senescence either as an inducer, marker, or consequence of senescent cells. We therefore place cellular senescence at the center as the most influential hallmark

In order to examine possible mechanisms, definition of criteria to determine hallmarks of aging is critical. A landmark report has classified nine hallmarks of aging based on three criteria: (a) the hallmark should manifest during normal aging; (b) its experimental augmentation should accelerate aging; and (c) its experimental attenuation should hamper normal aging, thus increasing healthy lifespan (Lopez‐Otin, Blasco, Partridge, Serrano, & Kroemer, 2013). These hallmarks are genomic instability, telomere attrition, epigenetic alterations, stem cell exhaustion, altered intercellular communication, loss of proteostasis, deregulated nutrient sensing, mitochondrial dysfunction, and cellular senescence. There is an intimate relationship between these hallmarks with fluctuations to one instigating changes in another. The most notable instance of this interconnectedness is with cellular senescence, a state of irreversible growth arrest coupled with stereotyped changes in phenotype and gene expression that represent all of the other hallmarks (Figure 1). In fitting with the above criteria, cellular senescence increases with age (Jeyapalan & Sedivy, 2008; Wang et al., 2009), and its augmentation and reduction, respectively, accelerate or diminish aging (Baker et al., 2016, 2011; Baker, Weaver, & van Deursen, 2013). Originally thought of as an in vitro phenomenon, senescent cells are increasingly thought to have a physiological role in age‐associated pathology (Campisi & Robert, 2014; Munoz‐Espin & Serrano, 2014).

As studies concerning the role of cellular senescence in age‐related disorders become increasingly common, senescence in the CNS is emerging as a new research topic. Taking into consideration that many neurodegenerative diseases including Alzheimer's disease (AD), Parkinson's disease (PD), and other types of dementia have age as a primary risk factor; the possibility that cellular senescence of CNS cell types may be a contributing factor can no longer be overlooked. The pro‐inflammatory secretory phenotype of senescent cells (described in more detail later) is particularly relevant to these diseases since inflammation and dysfunction of CNS cell types are key features of these disorders (Van Eldik et al., 2016; Tavazzi, Morrison, Sullivan, Morgello, & Fischer, 2014; Yan et al., 2014). Of the CNS cells, astrocytes are potential candidates for involvement in neurological disorders given their myriad roles in the maintenance of brain homeostasis. Senescence‐associated dysfunction of these glia would likely have profound effects in the contained environment of the CNS. In this review, we describe cellular senescence, its link to astrocyte physiology, astrocyte senescence, and their link to neurological disorders.

## 2 CELLULAR SENESCENCE

Cellular senescence is an age‐associated phenomenon first discovered in vitro after extensive culturing of fibroblasts (Hayflick, 1965). This phenotype eventually became attributed to telomere attrition after successive rounds of DNA replication (Bodnar et al., 1998). Senescence was later found to be induced prematurely in response to numerous internal and external stressors. Activation of tumorigenic signals including Myc and oncogenic RAS can lead to oncogene‐induced senescence (OIS) (Serrano, Lin, McCurrach, Beach, & Lowe, 1997). Alternatively, cytotoxic stimuli such as oxidative stress and proteasome inhibition can result in stress‐induced premature senescence (SIPS) (Bitto et al., 2010; Torres, Lewis, & Cristofalo, 2006). With the exception of telomere attrition (Harley, Futcher, & Greider, 1990) which is specific for replicative senescence, there are several phenotypes shared among senescent cells including cell cycle arrest and expression of cell cycle inhibitors p21 and p16 (Herbig, Jobling, Chen, Chen, & Sedivy, 2004), increased heterochromatin foci (Kosar et al., 2011; Kreiling et al., 2011), DNA damage foci (Rodier et al., 2011; Takai, Smogorzewska, & de Lange, 2003), increased senescence‐associated beta‐galactosidase (SA‐β Gal) activity (Dimri et al., 1995), enlarged and flattened cell morphology (Hayflick, 1965), decreased lamin B1 expression (Freund, Laberge, Demaria, & Campisi, 2012), and the secretion of pro‐inflammatory chemokines, cytokines, proteases, and growth factors, a phenomenon known as the senescence‐associated secretory phenotype (SASP) which can be regulated by NF‐κB (Coppe et al., 2008; Rodier et al., 2009). There is a large amount of heterogeneity to these phenotypes with their expression changing based on the cell type and senescence‐inducing stimulus. It is typical for multiple markers to be used for probing cellular senescence and while not all markers are specific, permanent growth arrest and SA‐β Gal positivity are generally present in senescent cells.

Of these senescence markers, the SASP may be the most influential due to the increased tissue inflammation associated with aging mammals known as “inflammaging” (Franceschi et al., 2007). Since senescent cells are known to increase with age, it is likely that the pro‐inflammatory secretions from these cells contribute to heightened inflammation in the elderly. Additionally, inflammation has been associated with numerous disorders ranging from cardiovascular to neurological (Jabbari Azad et al., 2014; Jenny et al., 2012), which suggests that cellular senescence and SASP may play a role in these and other diseases as well.

There are two main mechanisms put forth to account for cellular senescence leading to pathology in vivo. One is a cell autonomous process that reduces the pool of cycling cells available to an organism (van Deursen, 2014). This is thought to disrupt an organism's regenerative capacity and tissue homeostasis by depleting available stem and progenitor cells as well as impairing the stem cell niche. Some evidence for this comes from the fact that BubR1 progeroid mice used to study aging have progenitor cells from fat and skeletal muscle tissue that are highly prone to senescence (Baker et al., 2013). Additionally, clearance of senescent cells from aged mice reduced the number of adipose progenitors in white adipose tissue compared to mice without senescent cell clearance (Baker et al., 2016), providing support for this cell autonomous model.

Nonautonomously, senescent cells secrete myriad pro‐inflammatory cytokines and proteases which can adversely affect the surrounding microenvironment. These modifications involve stimulation of tissue fibrosis (Laberge, Awad, Campisi, & Desprez, 2012), perturbation of tissue structure by the cleavage of extracellular matrix proteins and surface receptors (Coppe et al., 2008), compounding cell autonomous effects via agitation of the stem cell niche (Pricola, Kuhn, Haleem‐Smith, Song, & Tuan, 2009), and paracrine senescence by induction of senescence in healthy neighboring cells (Acosta et al., 2013). It is important to note that these nonautonomous functions can be helpful in the short term, especially in the case of wound healing (Campisi & Robert, 2014). However, senescent cells are resistant to apoptosis (Wang, 1995) and therefore must be cleared by immune cells. In the long term, failure to clear these cells can add up as a contributor to the overall deterioration experienced in aging organisms (Chinta et al., 2014). The fact that a mouse model of low‐grade chronic inflammation demonstrated increased senescent cells and accelerated aging (Jurk et al., 2014) provides further support for cell nonautonomous contributions of senescent cells with aging.

Taking into consideration these deleterious effects, the role of senescence in the brain should be examined further. There are numerous cell types in the CNS ranging from neurons to glia which all have specialized functions. It is easy to imagine that dysfunction of one or more of these cells due to senescence could have far reaching pathological effects on tissue homeostasis and disease. One CNS cell type that is increasingly relevant is astrocytes which play a large role in healthy brain physiology and homeostasis.

## 3 ASTROCYTES

Human astrocytes comprise ~20% of glia with varying ratios to neurons depending on the CNS region (von Bartheld, Bahney, & Herculano‐Houzel, 2016; Herculano‐Houzel, 2014). Astrocytes are extremely heterogeneous and known to be necessary for a wide variety of vital functions in CNS physiology (Sofroniew & Vinters, 2010); human astrocytes in particular have a functional complexity and structural diversity allowing the human brain to be distinguished from that of other species (Oberheim, Wang, Goldman, & Nedergaard, 2006). This diversity has led to the classification of subtypes based on their morphology and anatomical location including (but not limited to) protoplasmic astrocytes found in gray matter, fibrous astrocytes found in white matter, interlaminar astrocytes, radial astrocytes, velate astrocytes of the cerebellum, cerebellar Bergmann glial, pituicytes of the neuro‐hypophysis, perivascular astrocytes, Gomori‐positive astrocytes of the hypothalamus and hippocampus as well as many others (Verkhratsky, Zorec, & Parpura, 2017). In addition, a recent study of hippocampal and striatal astrocytes found major differences in their electrophysiological properties, astrocyte–synapse proximity, and Ca^2+^ signaling, suggesting that astrocytes can differ based on their neural circuits (Chai et al., 2017).

Despite heterogeneity of astrocytic subtype, these glia have a far reaching anatomical organization with a presence throughout the entire CNS (Nedergaard, Ransom, & Goldman, 2003). It is thus clear that astrocytes are essential for CNS homeostasis (please see Verkhratsky & Nedergaard, 2018 for an in‐depth summary of astrocytic function). Curiously, glial‐specific gene expression patterns alter with age while relatively little occurs in neurons (Soreq et al., 2017), suggesting that aging could have a profound effect on astrocyte function. In fact, aging increases the expression of glial fibrillary acidic protein (GFAP) and vimentin (Porchet et al., 2003), two proteins associated with astrocyte activation. Supporting this, astrocytes from aged mice display an A1‐like reactive neuroinflammatory phenotype (Clarke et al., 2018). A1 reactive astrocytes are extremely neurotoxic and are unable to carry out their normal functions, impairing synapse formation (Liddelow et al., 2017). A reactive phenotype from aging and senescence may therefore contribute to neuropathology. In the next section, we define reactive astrocytes in more detail and describe its relationship to cellular senescence.

### 3.1 Astrogliosis

It is known that in response to insults and diseases of the CNS, astrocytes undergo profound structural changes in a phenomenon termed astrogliosis. There have been many attempts to define this phenomenon. Based on numerous studies in animal models and human pathology, a working definition of astrogliosis has been put forth based on four key features: “(a) astrogliosis is a spectrum of potential molecular, cellular, and functional changes in astrocytes that occur in response to all forms and severities of CNS injury and disease; (b) changes undergone by reactive astrocytes vary with severity of the insult along a graded continuum; (c) changes associated with astrogliosis are regulated in a context‐specific manner by many different inter‐ and intracellular signaling molecules; and (d) changes undergone during astrogliosis have the potential to alter astrocyte activities both through gain and loss of functions” (Sofroniew & Vinters, 2010). Based on these key features, astrogliosis is not an all‐or‐none event but a varying phenomenon. Even though astrogliosis is a continuous spectrum, it can be broadly categorized into three levels of severity for descriptive purposes.

The first‐level, mild‐to‐moderate astrogliosis is characterized by changes in gene expression that includes upregulation of GFAP, an intermediate filament protein which serves as a canonical marker of astrogliosis (Sofroniew, 2014). This rise in GFAP can sometimes lead to a false impression of increased proliferation. However, no proliferation changes are present in mild astrogliosis. Additionally, there is hypertrophy of the cell body and processes without any substantial changes to astrocytic domains and overlap. Depending on the extent of the injury, mild‐to‐moderate astrogliosis can resolve back into healthy tissue.

Severe diffuse astrogliosis has much of the same features as above including increased GFAP expression and cellular hypertrophy (Sofroniew, 2014). However, this level of astrogliosis includes increased proliferation and overlapping of astrocytic processes, disrupting individual domains. The ultimate result is a long‐lasting remodeling of tissue architecture that extends diffusely (hence the name) over large regions that do not resolve.

Severe astrogliosis with compact glial scar formation once again shares many features with its lesser forms, the main difference being the intensity of the phenotypes (Sofroniew, 2014). There is pronounced overlap of astrocytic processes and complete destruction of individual domains. The most prominent feature is the formation of dense, compact glial scars that are thought to occur in part by extensive escalation in astrocyte proliferation (Bardehle et al., 2013). These scars form alone the borders of severe CNS insults and infections as a neuroprotective barrier (Wanner et al., 2013). Interestingly, glial scars can interface with other cell types at the site of injury (Herrmann et al., 2008). Due to the extensive tissue reorganization that occurs as a result of this scarring, structural changes tend to be permanent, even after resolution of the initiating factor.

Importantly, GFAP alone is not an ideal marker of reactivity due to it being found at varying levels in resting astrocytes. Instead, markers of reactivity are more recently becoming dependent on injury and disease. For example, reactive astrocytes had different gene expression profiles dependent on whether they were from a model of stroke or neuroinflammation (Zamanian et al., 2012). Lcn2 and Serpina3n were also identified as shared markers of reactive astrocytes between these two models. Another study characterized a type of highly neurotoxic reactive astrocytes termed A1 astrocytes and found within this subtype that the complement component 3 (C3) gene is highly upregulated while not present in A2 ischemic astrocytes (Liddelow et al., 2017). Importantly, the authors found that C3 positivity overlapped with Alzheimer's disease, Huntington's disease, Parkinson's disease, amyotrophic lateral sclerosis, and multiple sclerosis, providing further support for reactive astrocytes markers being context dependent.

Independent of what markers are used, one common feature of reactive astrocytes is the release of numerous effector molecules such as cytokines, chemokines, and proteases (Sofroniew, 2014). These secretions can cause both pro‐ and anti‐inflammatory effects on the CNS, suggesting that reactive astrocytes can have both detrimental and positive effects on the CNS. For example, reactive astrocytes have been shown to cause synaptic dysfunction, impair neural outgrowth, and induce neuronal cell death (Liddelow et al., 2017). Harmful factors secreted from reactive astrocytes can even exacerbate inflammation that occurs in response to injury (Eddleston & Mucke, 1993; Spence et al., 2013). On the beneficial side, a transgenic model which ablates astrogliosis led to increased inflammation (Wanner et al., 2013). More recently, ablation of reactive astrocytes from glial scars was found to be detrimental to axon growth and repair (Anderson et al., 2016), highlighting that astrogliosis may have far reaching benefits to the point where simply removing reactive astrocytes would not be a viable therapy for neurodegeneration. In fact, the question of how astrocytes become reactive is an important point when it comes to targeting cognitive decline. Since astrocytes have been shown to become reactive in response to active microglia (Clarke et al., 2018; Liddelow et al., 2017) and microglia become activated with age (Mosher & Wyss‐Coray, 2014), simply targeting astrocytes may not be enough to intervene neurocognitive diseases.

**Figure 2:**
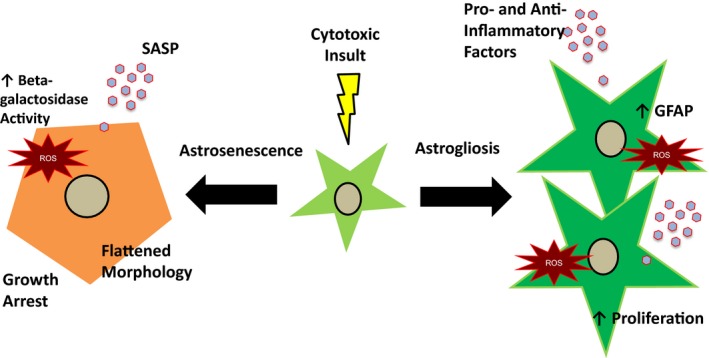
Comparison of astrocyte senescence (astrosenescence) and astrogliosis. In response to stress, there are several possible pathways that an astrocyte may undergo. With astrogliosis, an astrocyte becomes hypertrophic with enlargement of the cell body and processes. There is an upregulation of the intermediate filament GFAP and the secretion of many pro‐ and anti‐inflammatory cytokines, chemokines, and growth factors. With more severe astrogliosis, there is also increased proliferation and overlap of astrocytic domains. Astrocyte senescence involves growth arrest and enlargement with flattened cell morphology. Senescent astrocytes have increased lysosomal mass which results in increased beta‐galactosidase activity and they have a pro‐inflammatory profile of cytokines, chemokines, and growth factors that can be controlled by NF‐κB known as the senescence‐associated secretory phenotype

One overlooked aspect is that there is a great deal of overlap between the secretions of reactive astrocytes and the SASP of senescent cells. In fact, cellular senescence shares many of the same features as astrogliosis including cellular enlargement and pro‐inflammatory secretion, raising the possibility that many studies, which looked at reactive astrocytes, may have actually been examining senescence. This is supported by the fact that aged astrocytes take on a reactive phenotype associated with neuroinflammation (Clarke et al., 2018). There are now enough distinctions between astrogliosis and senescence to allow identification (Figure 2 and Table 1) and as such coin the cellular changes that a senescent astrocyte undergoes “astrosenescence.” Since astrocytes in the aged brain display characteristics of the SASP (Bhat et al., 2012; Clarke et al., 2018), a much greater emphasis should be placed on the role of astrosenescence in response to CNS injury and disease. Indeed, old astrocytes isolated from a mouse model of AD have increased levels of IL‐6 compared to WT (Iram et al., 2016). Astrocytes in rats treated with Aβ‐oligomers demonstrated marked inflammation associated with NF‐κB (Carrero et al., 2012). Since astrocyte inflammation is associated with neurodegenerative disease, it is highly likely that astrosenescence plays a contributing role. In the next section, we provide a comprehensive overview of studies to date, which examined astrocyte senescence from in vitro to in vivo.

**Table 1:** Features and markers of cellular senescence and astrogliosis

Phenotypic changes	Senescence	Senescence‐associated markers		Astrogliosis	Astrogliosis associated markers	
Structural	Flattened cell morphology	Large and rounded cells	Hayflick (1965) Bhat et al. (2012)*, Cohen et al. (2017)*, Souza et al.( 2013), Yu et al. (2017)*	Cellular Hypertrophy	Enlarged cell body and processes	Sofroniew (2014)
				Intermediate Filament Upregulation	Increased GFAP	Eddleston and Mucke (1993)
Cell cycle changes	Growth arrest	p16	Serrano et al. (1997) Bhat et al. (2012)*, Evans et al. (2003)*, Yu et al. (2017)*	Increased proliferation	JNK/c‐Jun, scar formation	Bardehle et al. (2013)
		p21, p53	Herbig et al. (2004) Bhat et al. (2012)*, Cohen et al. (2017)*, Evans et al. (2003)*, Turnquist et al. (2016)*			
		Telomere attrition	Harley et al. (1990) Evans et al. (2003)*			
Nuclear changes	Heterochromatin rearrangements	mH2A, HP1	Kreiling et al. (2011)	Nuclear translocation of transcription factors	STAT3	Herrmann et al. (2008)
		H3K9me3	Kosar et al. (2011)			
		HIRA	Bitto et al. (2010)*			
	DNA damage response	yH2AX, 53BP1	Rodier et al. (2011)			
		Telomere dysfunction‐induced foci	Takai et al. (2003)			
	Nuclear lamina changes	Decreased Lamin B1	Freund et al. (2012)			
Lysosomal changes	Increased lysosomal mass	Senescence‐associated β‐galactosidase	Dimri et al. (1995) Bhat et al. (2012)*, Bitto et al., (2010)*, Cohen et al. (2017)*, Evans et al. (2003)*, Yu et al. (2017)*	Unknown		
Inflammation	Senescence‐associated Secretory Phenotype	IL‐6, IL‐8, IL‐1, MMPs, etc	Acosta et al. (2008), Coppe et al. (2008); Bhat et al. (2012)*, Cohen et al. (2017)*	Cytokines and growth factors	IL−6, IL−1, TGFβ, etc	Sofroniew (2014)

Note

Asterisk in Senescence‐associated markers column indicates astrocyte senescence citation.

## 4 ASTROCYTE SENESCENCE

Early studies have demonstrated that CNS tissue could grow in vitro (Buckley, 1929; Kredel, 1928), and subsequent studies expanded these techniques as a means to observe morphology from histologic sections to the level of a single living cell (Costero, Pomerat, Jackson, Barroso‐Moguel, & Chevez, 1955; Manuelidis & Pond, 1959; Shein, 1965, 1967).

The first study to look at the effect of long‐term culturing on CNS cells compared properties of glial cell lines isolated from both benign and malignant origin (Ponten & Macintyre, 1968). Based on morphological characteristics, these glia were identified as having the properties of astrocytes. The study found that while some of the cells isolated from gliomas were able to be cultured indefinitely, cells isolated from normal tissue had a finite lifespan that terminated after a set number of passages. Though it was not described in those terms at the time, this was the first study to document replicative senescence in an astrocytic cell line.

The previous study was expanded on using a technique known as minicloning in which “islands” of palladium surrounded by agarose were created (Blomquist, Westermark, & Ponten, 1980). When plated, cells were only able to attach to the “islands” and could not expand to the surrounding agarose. By looking at the “islands” that only had one cell, it was possible to track growth over time by counting the number of cells in the region. It was found that glial cells from later passages had a reduced capacity for population doublings, demonstrating a relationship between growth capacity and cellular age. These senescent cells, termed “nondividers” at the time, were predominantly arrested at the G_1_ phase of the cell cycle, indicating that this arrest happened before any errors associated with DNA replication.

Two models of cell aging had been put forward to describe the growth kinetics of cellular senescence. The first model proposed that senescence is merely an artifact of tissue culture (Holliday, Huschtscha, Tarrant, & Kirkwood, 1977). Within a cell population, there are a proportion of nonsenescent cells with an infinite doubling potential. Over time, these cells become diluted and lost due to serial subcultivations and when they are completely depleted, one is left with nondividing cells. This theory predicts that the proportion of nondividing cells remain constant in a culture until the cells with infinite growth potential are discarded by chance.

The second model of cellular aging proposed that senescence is a function of population doublings, namely that the probability of mortalization, that is, senescence, increases from zero to one with rising numbers of cell cycles (Shall & Stein, 1979). It predicts that the fraction of nondividing cells should multiply over time with serial passaging as long as it is not selective for or against cells with a potential for further division. However, this model does not predict the number of population doublings at which the increase in the number of senescent cells first becomes evident.

In order to determine which of the two models best fits the glial growth pattern initially observed by Blomquist (Blomquist et al., 1980), a follow‐up study was performed that more specifically examined growth kinetics (Ponten, Stein, & Shall, 1983). Using the miniclone method, it was found that while at early passages, only a small number of glia were unable to divide and produce colonies, at later passages this number gradually increased. Additionally, colony size decreased with increasing population doublings. The data suggest that it is no catastrophe which causes a sudden change in the senescent cell population; instead, there is a gradual and constant increase in nondividing cells that most closely fits the Shall and Stein model described above (Shall & Stein, 1979). Most notably, the growth patterns for these glia match that of fibroblast senescence (Hayflick, 1965).

With time, better methodologies were developed for the extraction of specific cell types and senescence was studied using primary astrocyte cultures. One such study used senescence of primary human astrocytes in order to examine mechanisms that control proliferative lifespan (Evans, Wyllie, Wynford‐Thomas, Kipling, & Jones, 2003). Astrocytes that underwent replicative senescence displayed classical markers of senescence including senescence‐associated β‐galactosidase activity, p21 expression, and a growth arrest dependent on p53. Intriguingly, this initial p53‐dependent replicative senescence was independent of telomere erosion and it was only after escape of a second, p16‐dependent checkpoint that telomerase expression led to immortalization. The authors thus proposed that these senescence checkpoints need to be bypassed for tumor formation. Alternatively, it was demonstrated that astrocytes isolated from postmortem AD patients displayed increased levels of the senescence markers p16, p21 and eventual growth arrest at late passages of in vitro culturing compared to early passages once again demonstrating replicative senescence from primary astrocytes (Blasko et al., 2004). Unfortunately, no replicative senescence comparison was made between AD and age‐matched controls.

To evaluate the role of replicative senescence on brain function, rat astrocytes made senescent by in vitro culturing for 90 days, showed signs of stress and glutamate uptake as well as decreased mitochondrial activity (Pertusa, Garcia‐Matas, Rodriguez‐Farre, Sanfeliu, & Cristofol, 2007). Importantly, when senescent astrocytes were co‐cultured with neurons, there was reduced neuronal survival compared to nonsenescent astrocytes, demonstrating that astrocyte senescence can cause neuronal death. Similarly, senescent mouse astrocytes also had adverse effects on co‐cultured neurons (Kawano et al., 2012). These co‐cultured neurons had decreased glutamate release from the synapse and an overall reduction in the available pool of synaptic vesicles. This reduction is likely due in part to retarded synapse maturation in these neurons.

Neurons are not the only CNS cell type shown to be adversely affected by aged astrocytes. Conditioned media from aged astrocytes, isolated from 13‐month‐old mice, caused a reduction in neural progenitor cells (NPC) proliferation compared to NPC incubated with conditioned media from young astrocytes, isolated from 3‐month‐old mice (Miranda et al., 2012). This growth deficit was tied to decreased Wnt production in aged astrocytes which in turn diminished expression of the anti‐apoptotic protein survivin in NPCs. These results suggest that astrocyte senescence could affect neurogenesis during aging.

Astrocytes have also been documented to undergo stress‐induced premature senescence. One of the first studies to characterize this looked at the senescence response of mouse and human astrocytes treated with H_2_O_2_ or the proteasome inhibitors lactacystin and epoxomicin (Bitto et al., 2010). Exposure to these stressors induced a senescence phenotype that included decreased proliferation, increased senescence‐associated β‐galactosidase, and expression of senescence markers p16, p21, and p53. Interestingly, astrocytes in this study were found to be more sensitive to senescence‐inducing stimuli than fibroblasts, suggesting that there may be differences in the senescence program based on cell type or even the senescence‐inducing stimulus.

One example of these differences can be found in a study that compared H_2_O_2‐_treated rat astrocytes with astrocytes isolated from old animals (Souza, Bellaver, Souza, & Quincozes‐Santos, 2013). While there have been similarities in phenotype to replicative senescence such as an increase in ROS and cellular enlargement, aged cells from old animals had increased glutamate uptake (Pertusa et al., 2007) while H_2_O_2_ caused a decrease (Souza et al., 2013). The ROS and morphology phenotypes were reversed by treatments with resveratrol and a p38MAPK inhibitor, suggesting that senescence could be targeted therapeutically. Indeed, ammonia‐induced senescence of rat astrocytes demonstrated attenuation of p53‐mediated senescence through inhibitors of glutamine synthetase, ROS, and p38MAPK (Gorg, Karababa, Shafigullina, Bidmon, & Haussinger, 2015). ROS inhibition also prevented angiotensin II induced astrocyte senescence, suggesting therapeutic potential for cerebral ischemic injury (Liu et al., 2011).

Notably, the timing of these therapeutics should be taken into consideration when used to alleviate senescence. Metabolites of ginseng known as ginsenosides, which are thought to have anti‐aging properties, could prevent H_2_O_2_‐mediated senescence of rat astrocytes and a human astroglioma cell line cells but were unable to reverse senescence after the fact (Hou, Kim, Sung, & Choi, 2017). However, pro‐inflammatory components of the SASP were reversed by ginsenosides after oxidative stress‐induced senescence, suggesting that some therapeutics may still be able to ameliorate negative effects associated with senescence.

Thus, astrocytes have been shown to undergo cellular senescence in vitro due to a variety of factors ranging from replicative lifespan to external stressors. There is some debate as to the physiological relevance of this phenomenon given that terminally differentiated cells are not thought to proliferate in vivo. However, an immunostaining analysis of human CNS biopsy and autopsy tissues using proliferation marker MIB‐1 found evidence of low‐level astrocyte proliferation in vivo (Colodner et al., 2005 *)*. Studies of aging biomarkers found that p16, a key indicator of senescence, increases in aging cortices, demonstrating in vivo evidence of CNS senescence during aging (Bhat et al., 2012; Krishnamurthy et al., 2004). Remarkably a significant population of senescent astrocytes overexpressing p16 is present in the frontal cortex of AD patients (Bhat et al., 2012). Considering the importance of astrocytes to normal CNS homeostasis and the fact that there are vast changes to the transcriptome of senescent astrocytes (Crowe et al., 2016), it is likely that astrocyte senescence has a severe impact on neurodegenerative diseases. In the next section, we describe several neurological disorders and emerging evidence that astrocyte senescence may contribute to their pathology.

## 5 NEURODEGENERATIVE DISEASE AND ASTROCYTE SENESCENCE

Alzheimer's disease (AD) is a neurological disorder that causes problems with memory, behavior, and executive functions. It is the most common type of dementia, accounting for nearly 60%–80% of all cases. An estimated 5.5 million people have AD in the United States affecting 1 out of every 10 adults over 65 years old making it highly age‐associated. The pathophysiology of AD has been extensively studied leading to several hypotheses over the years. These are the cholinergic hypothesis, tau hypothesis, inflammation hypothesis, and amyloid‐β (Aβ) hypothesis. The Aβ hypothesis has been the most extensively studied. In this model, the amyloid precursor protein (APP) gets aberrantly cleaved by β and γ‐secretases instead of α‐secretase, resulting in an imbalance between the clearance and production of Aβ peptides. These peptides aggregate to form soluble oligomers, which eventually coalesce into insoluble plaques. These oligomers and plaques have been linked to neurofibrillary tangles, loss of synapse function, cerebrovascular damage, and glia activation, leading to neurotoxicity and cognitive decline (Kumar, Singh, & Ekavali, 2015). While therapeutics targeting Aβ do exist, their effects on slowing cognitive decline have been moderate at best, suggesting that other mechanisms may be involved. As astrocytes have been implicated in AD (Gonzalez‐Reyes, Nava‐Mesa, Vargas‐Sanchez, Ariza‐Salamanca, & Mora‐Munoz, 2017; Perez‐Nievas & Serrano‐Pozo, 2018), one alternative mechanism may involve astrocytic neuroinflammation since Aβ can activate pro‐inflammatory signaling in astrocytes and inflammation has been associated with AD severity (Jabbari Azad et al., 2014). Indeed, ablation of microglia in an AD mouse model did not affect plaque formation but astrocytes in this model remain active, suggesting that astrocytic inflammation is involved (Grathwohl et al., 2009). In addition, since one component of cellular senescence is the production of pro‐inflammatory cytokines known as the SASP (Campisi & Robert, 2014), a study demonstrating that astrocytes surrounding Aβ plaques are positive for the SASP component IL‐6 (Benzing et al., 1999) lends support for astrocyte senescence as a novel mechanism associated with AD pathogenesis. The first study to demonstrate a link between astrocyte senescence and AD treated human astrocytes in vitro with either conditioned media from Aβ‐producing CHO cells or the Aβ_1–42_ oligomer (Bhat et al., 2012). Treated astrocytes displayed classic signs of cellular senescence including a flattened morphology, increased p16 expression, and increased SA‐β galactosidase activity. Most profoundly, when frontal cortex tissue was examined for astrocyte senescence, not only was there an age‐associated increase in p16‐positive astrocytes, there was a further increase in AD patients compared to their age‐matched controls. Since there was a parallel cortex increase in the SASP component MMP‐1, and little astrocyte senescence in the cerebellum which has low AD pathology, this study demonstrates that astrocyte senescence may be a contributor to AD via their effect on the microenvironment. The authors also include in vitro evidence that the SASP may be ameliorated by targeting the p38MAPK pathway. A recent study has confirmed an increase in p16‐positive astrocytes in AD patients, demonstrating that brain homogenates contained increased mRNA of several other senescence markers including p21 and IL‐6 (Turnquist et al., 2016). These authors also looked at two p53 isoforms and found that Δ133p53 was downregulated while p53β was upregulated in AD patients, matching a pattern found with astrocyte senescence in vitro. Overexpression of Δ133p53 not only reduced astrocyte senescence, it also prevents neuronal death associated with co‐culture of senescent astrocytes. Thus, astrocyte senescence associated with p53 isoforms may be another therapeutic target for AD.

Parkinson's disease (PD) is the second most common progressive neurodegenerative disorder in the United States, affecting 2%–3% of adults over 65. Typically considered an age‐associated disease, the prevalence in older Americans is only expected to increase. PD is characterized as a disorder that mainly affects the motor system. Classic symptoms include a resting tremor that disappears with voluntary movement, bradykinesia, rigidity, and postural instability. Later stages of the disease can include dementia. Pathologically, PD is defined by death of the dopaminergic neurons in the basal ganglia's substantia nigra. This is thought to happen in part by the abnormal accumulation of alpha‐synuclein inside neurons, forming protein aggregates known as Lewy bodies (Beitz, 2014). These aggregates can impair normal neuronal function, leading to neurodegeneration. The exact triggering mechanism for PD is not yet known. While genetics may play a role due to family history being a risk factor, the majority of cases are spontaneous, suggesting that there could be environmental triggers. There are several environmental neurotoxic compounds now thought to induce PD, including the heroin analog 1‐methyl‐4‐phenyl‐1, 2,3,6‐tetrahydropyridine (MPTP) and the herbicides paraquat and 2,3,7,8‐tetrachlorodibenzodioxin (TCDD) (Gonzalez‐Barbosa et al., 2017). Since PD patients share many aspects of cellular senescence including inflammation (Yan et al., 2014), oxidative stress (Dias, Junn, & Mouradian, 2013), and mitochondrial dysfunction (Jiang, Jiang, Zuo, & Gu, 2013) and astrocytes have been shown to senesce in response to various stressors, it has been proposed that astrocyte senescence in response to environmental toxins could be a contributor to PD (Chinta et al., 2013). Indeed, astrocytes treated with TCDD underwent premature senescence with growth arrest accompanied by cytoskeletal remodeling and increases in p16, p21, and SA‐β gal (Nie et al., 2015). Since this phenotype included an increase in ROS levels, it was thought that TCDD might be inducing astrocyte senescence via oxidative stress. Interestingly, while ROS inhibition attenuated senescence, it was found that ROS was induced upstream by a WNT/β‐catenin signaling mechanism. Bearing in mind that ROS was also involved in TCDD‐mediated senescence of neuronal cells, antioxidants to prevent CNS cell senescence may be a potential treatment for PD. Most importantly, a recent publication demonstrated that PD patients had high levels of astrocytes positive for senescence markers (Chinta et al., 2018). Treatment with paraquat induced astrocyte senescence both in vitro and in mice. Moreover, depletion of senescent cells in these mice diminishes paraquat‐mediated PD‐like neuropathological phenotypes. Consequently, the role of astrocyte senescence in PD should therefore be strongly considered.

HIV‐1 infection occurs worldwide, affecting nearly 36 million people and 1 million in the United States alone. Improvements to highly active antiretroviral therapy (HAART) has resulted in an aging HIV‐infected population, and at the current rate, the proportion of HIV‐infected individuals 50 years old or higher will reach 73% by 2030 (Negredo et al., 2017). Intriguingly, the HIV‐infected population suffer from a variety of ailments experienced in the elderly; therefore, patients are sometimes described as undergoing accelerated aging (Guaraldi et al., 2011). One of these comorbidities, neurological problems associated with HIV known as HIV‐associated neurocognitive disorders (HAND), is particularly relevant to aging in the CNS. Characterized by cognitive impairment, motor dysfunction, speech problems, and behavioral changes, the exact cause of HAND in the post‐HAART era is not yet known (Saylor et al., 2016). Studies suggest dysfunction due to viral reservoirs in the CNS as well as toxicity from the HAART drugs themselves (Smith, de Boer, Brul, Budovskaya, & van Spek, 2012). Given that premature senescence from HAART drugs and HIV proteins has been linked to comorbidities in non‐CNS cell types (Beaupere et al., 2015; Nacarelli, Azar, & Sell, 2016), astrocyte senescence in response to these stimuli could be a contributor to HAND. Indeed, a very recent study demonstrated that human astrocytes infected with an HIV plasmid underwent premature senescence (Yu et al., 2017). Conditioned media from these senescent astrocytes induced neuronal apoptosis suggesting that senescent astrocytes could have an adverse effect on the CNS microenvironment in the context of HIV and HAND. Notably, HIV‐associated astrocyte senescence was mediated by the WNT/β‐catenin signaling pathway, matching results from other senescence‐associated disease models. The authors also demonstrated an increase in senescent astrocytes from several rat models of HIV infection, providing in vivo relevance. On the antiretroviral side of HIV‐associated stimuli, HAART drug has been shown to induce premature senescence in human astrocytes, the first study to demonstrate HAART‐mediated senescence of a CNS cell type (Cohen, D'Agostino, Wilson, Tuzer, & Torres, 2017). These astrocytes showed signs of oxidative stress and mitochondrial dysfunction accompanied by changes in metabolism, indicating that HAART drug‐mediated astrocyte senescence can dramatically affect normal astrocyte physiology. Importantly, these cells show signs of increased pro‐inflammatory signaling, suggesting that there could be a pro‐inflammatory microenvironment from senescent astrocytes in HAND patients. CNS inflammation is found in HAND patients (Tavazzi et al., 2014), and the role of astrocyte senescence in HAND pathogenesis should be examined further.

## 6 CONCLUSIONS AND UNRESOLVED QUESTIONS

**Figure 3:**
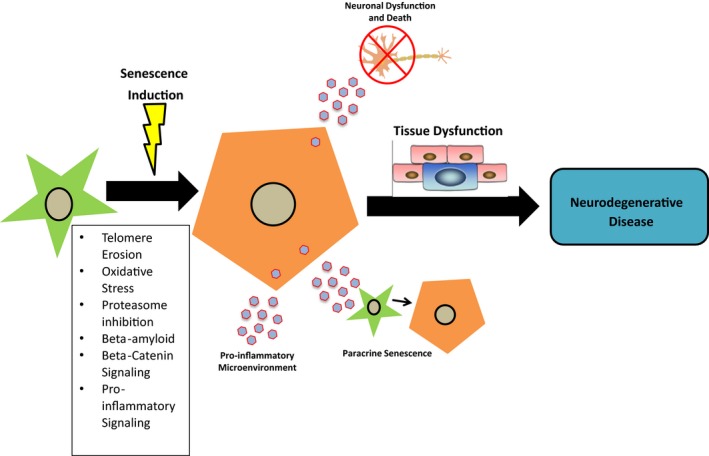
Astrocyte senescence as a contributor to neurodegenerative disease. After exposure to stressors such as telomere erosion, oxidative stress or inflammation an astrocyte may undergo cellular senescence. The senescent phenotype can induce adverse effects on the local CNS microenvironment including neuronal dysfunction and death, paracrine senescence of neighboring cells, and inflammation. These factors can contribute to CNS tissue dysfunction, leading to neurodegenerative disease

Astrocytes have been shown to undergo replicative cellular senescence in vitro and can senesce prematurely in response to various stressors. In vivo, senescent astrocytes have been shown to accumulate with age and in the context of neurological disorders. The cellular changes associated with this phenomenon can be collectively termed “astrosenescence.” The detrimental impact these cells could contribute to the tissue microenvironment suggests that astrosenescence may contribute to the pathology of age‐associated neurological diseases (Figure 3).

Within a senescent cell, there can be various disruptions to normal cellular physiology including increases in ROS, mitochondrial dysfunction, and inflammation. Notably, these are features also associated with neurodegenerative disorders such as AD (Jabbari Azad et al., 2014; Kumar et al., 2015), PD (Chinta et al., 2013), and HAND (Fields et al., 2016; Tavazzi et al., 2014). Targeting these phenotypes may be an important line of treatment in patients affected by these diseases and promising results are already shown in several disease models (Choi et al., 2014; Deshpande et al., 2005).

An alternative line of therapy for the treatment of these disorders may be the clearance of senescent cells. This concept has been demonstrated with great success in transgenic mice that express constructs capable of inducible senescent cell clearance in order to extend healthy lifespan and reduce the effects of several age‐associated disorders (Baker et al., 2016, 2013; Jeon et al., 2017). Most recently, this concept has successfully been tested in a mouse model of tau‐dependent neurodegeneration (Bussian et al., 2018). Mice in this study accumulate senescent astrocytes and microglia, clearance of which prevents tau deposition and degeneration of cortical and hippocampal neurons, the very first study to demonstrate a causal link between glial senescence and neurodegeneration. In humans however, using such approach may not be possible. Instead, a similar effect might be conceivable using a new class of drugs known as senolytics (Kirkland, Tchkonia, Zhu, Niedernhofer, & Robbins, 2017). These pharmacological agents work by selectively inducing apoptosis in senescent cells and have already been successful in preclinical trials of several age‐associated diseases ranging from cardiovascular to osteoporosis (Roos et al., 2016; Zhu et al., 2015). The previous study of tau‐dependent neurodegeneration also demonstrated therapeutic potential with senolytic treatment, suggesting that senolytics to clear senescent astrocytes could be beneficial to age‐associated neurogenerative diseases (Bussian et al., 2018). Alternatively, interventions that delay aging, the senescence program, and SASP, represent meaningful approaches to evaluate the role of modulation of senescence on neurocognitive diseases earlier stages as aging progress. However, preclinical studies require the previous assessment of the extent of senescence in the CNS of available animal models.

The possibility that senescence of other CNS cell types could play a role in neurodegenerative disease should not be discounted. In fact, cellular senescence has been identified in microglia (Flanary, Sammons, Nguyen, Walker, & Streit, 2007), oligodendrocytes (Al‐Mashhadi et al., 2015), neural stem cells (He et al., 2013), and even neurons (Jurk et al., 2012). We have recently demonstrated that microglia can senesce in response to HIV infection and have proposed that microglia may transition from activation to senescence (Chen, Partridge, Sell, Torres, & Martin‐Garcia, 2017; Chen et al., 2018). Thus, the link between other CNS cell senescence and age‐associated neurodegenerative disease should be further explored.

Nevertheless, astrocyte senescence is an emergent field of study and more research needs to be performed to examine autonomous and nonautonomous mechanisms of senescent astrocytes and the link between astrocyte senescence and age‐associated neurodegenerative diseases. There are a number of potential disorders where the presence of senescent astrocytes has yet to be examined including Huntington's disease, frontotemporal dementia, Friedreich's, and HIV‐associated neurocognitive disorders. Additionally, the pathologies that do demonstrate increased senescent astrocytes have so far only shown correlation. Animal models of neurological disease should be combined with targeted treatments that reduce the number of senescent astrocytes in the CNS. The results from these future studies could potentially begin a new era in aging medicine.

## CONFLICT OF INTEREST

None declared.
